# Comparison of multiplicative and additive hyperbolic and hyperboloid discounting models in delayed lotteries involving gains and losses

**DOI:** 10.1371/journal.pone.0233337

**Published:** 2020-05-22

**Authors:** Wojciech Białaszek, Przemysław Marcowski, David J. Cox

**Affiliations:** 1 SWPS University of Social Sciences and Humanities, Warsaw, Poland; 2 JHUSOM Department of Psychiatry and Behavioral Sciences, Johns Hopkins Medicine, Odenton, Maryland United States of America; University of Leeds, UNITED KINGDOM

## Abstract

Many day-to-day decisions may involve risky outcomes that occur at some delay after a decision has been made. We refer to such scenarios as delayed lotteries. Despite human choice often involves delayed lotteries, past research has primarily focused on decisions with delayed or risky outcomes. Comparatively, less research has explored how delay and probability interact to influence decisions. Within research on delayed lotteries, rigorous comparisons of models that describe choice from the discounting framework have not been conducted. We performed two experiments to determine how gain or loss outcomes are devalued when delayed and risky. Experiment 1 used delay and probability ranges similar to past research on delayed lotteries. Experiment 2 used individually calibrated delay and probability ranges. Ten discounting models were fit to the data using a genetic algorithm. Candidate models were derived from past research on discounting delayed or probabilistic outcomes. We found that participants' behavior was best described primarily by a three-parameter multiplicative model. Measures based on information criteria pointed to a solution in which only delay and probability were psychophysically scaled. Absolute measures based on residuals pointed to a solution in which amount, delay, and probability are simultaneously scaled. Our research suggests that separate scaling parameters for different discounting factors may not be necessary with delayed lotteries.

## Introduction

In many non-laboratory situations, the choices we make might be influenced by the delay and probability of the consequences that follow. For example, a choice among financial investments is likely influenced by the delay to financial returns and the probability of receiving financial returns. Similarly, the choice to make a minor car repair may depend on the delay and probability that a larger issue will occur with the vehicle. Despite the seeming ubiquity of delayed and probabilistic outcomes, most decision-making research focuses on how just delay or just probability influences subjective value. Here, we investigate preferences involving outcomes that are both delayed and probabilistic–delayed lotteries.

Early approaches that investigated decision-making focused primarily on a choice between two outcomes occurring with different probabilities (risky choice) or between two outcomes occurring at different delays (intertemporal choice). Although many researchers pointed to parallels between risky and intertemporal choices from the outset, empirical work on the relationship between delay and probability began a few decades ago and has been comparatively scant [1–4]. Recently, however, this trend is changing with researchers developing a growing interest in discounting delayed lotteries [5–7] and other investigations of time-risk interactions [8–10].

One paradigm that describes how subjective value is influenced by specific characteristics of consequences is called discounting. Delay discounting refers to a decrease in the subjective value of an outcome as the delay to the outcome increases [11]. Probability discounting refers to a decrease in the subjective value of an outcome as the probability of contacting the outcome decreases [1]. Delay and probability discounting are well described in the domains of gains and losses by a simple hyperbola [12–15] that follows the equations [11]:

SV=A/(1+kD) (1A)

for delay, and

SV=A/(1+hθ) (1B)

for probability [1]. Where *SV* represents the subjective value of a gain or loss with a nominal value of *A*; *k* and *h* are free parameters that describe the rate that delay or probability reduces subjective value, respectively; and *D* stands for delay and *Ѳ* for the odds against the occurrence of a gain or loss (*Ѳ* = ((1 − *P*)⁄*P*); *P*, probability of contacting the outcome).

Research with human participants led to a modification of the simple hyperbolic function. The simple hyperbolic function often will systematically overpredict subjective value from humans at short delays and low odds against, and systematically underpredict subjective value from humans at long delays and high odds against [1,15,16]. To account for these deviations in model predictions, Green, Fry, and Myerson [16] raised the entire denominator of the simple hyperbolic function by a nonlinear scaling parameter (*s*) based on psychophysical power laws [17]. Later, Ostaszewski, Green, and Myerson [18] proposed the same modification to describe probability discounting data obtained from human participants. These hyperboloid equations take the following forms:

$$SV=A/{\left(1+kD\right)}^{s}$$ (2A)

for delay discounting, and:

$$SV=A/{\left(1+h\theta \right)}^{s}$$ (2B)

for probability discounting. Importantly, raising the entire denominator by *s* does not allow researchers to determine if psychophysical scaling occurs with *A* or *D* in delay discounting tasks [12] or with *A* or *Ѳ* in probability discounting tasks.

Another modification of the simple hyperbolic function was proposed by Rachlin [17]. Rachlin suggested adding the exponent *s* only to the independent variable–delay or odds against. In this formulation, Eqs 1A and 1B take the following form for delay and probability discounting, respectively:

$$SV=A/(1+kD^{s}),$$ (3A)

$$SV=A/(1+h\theta^{s}).$$ (3B)

Here, *s* represents nonlinear scaling specifically of delay or odds against. The advantage of this formulation is that researchers can easily compare individual differences in how participants scale changes in delays or odds against.

The simple hyperbola and hyperboloid equations above were designed to describe intertemporal or risky choices, not outcomes that involve both delay and probability. However, researchers have begun to explore how these simple equations can be combined to describe choice with delayed lotteries. Two types of discounting models have been proposed to account for choice with delayed and probabilistic outcomes. The first is an additive model which assumes that delay and probability influence subjective value independently. Specifically, the additive model assumes the subjective value of a delayed lottery is the nominal amount (*A*) minus the discounted amount from delay and minus the discounted amount from probability [7,19]. The two-parameter (the *k* and *h*) version of this model takes the form:

SV=A−A(1−1/(1+kD))−A(1−1/(1+hθ)). (4)

The second is a multiplicative model which assumes that delay and probability interact to influence outcome value [7,20]. The two-parameter version of this model takes the form:

SV=A/((1+kD)*(1+hθ)). (5)

The multiplicative model predicts that the influence of delay on outcome value will decrease as probability decreases, and the influence of probability on outcome value will decrease as delay increases. Finally, Eqs 4 and 5 reduce to the simple hyperbolic functions (Eqs 1A and 1B) when the outcome is only delayed or only probabilistic.

The ability for Eqs 4 and 5 to describe discounting with delayed lotteries has been examined only with human participants. Past researchers have found that the modified hyperbolic equations (Eqs 2A, 2B, 3A and 3B) describe human choice better than the simple hyperbolic functions (Eqs 1A and 1B). Thus, researchers studying Eqs 4 and 5 also explored various denominator exponentiated versions of the additive and multiplicative equations [7,21]. Two types of denominator exponentiated functions have been examined. First, the entire denominators in Eqs 4 and 5 can be raised to a single psychophysical scaling parameter (*s*) to make a three-parameter model (i.e., amount and independent variable scaled). Second, in an extension of the approach taken by Green, Myerson, and colleagues, researchers have raised the entire delay and the entire probability denominators to distinct psychophysical scaling parameters (*s*_*d*_ for delay, and *s*_*p*_ for probability) to make a four-parameter model. Finally, in an extension of the approach taken by Rachlin, one could exponentiate only the independent variables. That is, one could use a single psychophysical scaling parameter to exponentiate only the independent variables to make a three-parameter model; or, one could use a distinct psychophysical scaling parameter to make a four-parameter model. No researchers have examined how these Rachlin-esque models describe delayed lotteries.

Table 1 presents the eight formulations of discounting models for delayed lotteries described above. The structure of each model represents a unique combination of assumptions about the relationship between delay and probability, and assumptions about psychophysical scaling. Eqs 6–9 assume delay and probability independently influence final subjective value (i.e., “Additive”) whereas Eqs 10–13 assume delay and probability interact to influence final subjective value (i.e., “Multiplicative”). Eqs 6, 7, 10 and 11 assume that psychophysical scaling occurs relative to the amount and the independent variable (i.e., “Amount & IV Scaled”) whereas Eqs 8, 9, 12, and 13 assume psychophysical scaling occurs only relative to the independent variable (i.e., “Only IV Scaled”). Finally, Eqs 6, 8, 10, and 12 assume that all psychophysical scaling occurs similarly (i.e., “# of Parameters” equals “3”) whereas Eqs 7, 9, 11, and 13 assume that psychophysical scaling is dimension specific (i.e., changes based on outcome dimension; “# of Parameters” equals “4”).

**Table 1:** Mathematical formulations of three- and four-parameter additive and multiplicative discounting models.

Parameters	Model	Equation	Model formula
**Additive**			
Amount & IV scaled			
3	ADD3	(6)	$SV=A-A(1-1/{\left(1+kD\right)}^{s})-A(1-1/{\left(1+h\theta \right)}^{s})$
4	ADD4	(7)	$SV=A-A(1-1/{\left(1+kD\right)}^{sd})-A(1-1{\left(1+h\theta \right)}^{sp})$
Only IV scaled			
3	ADD3R	(8)	$SV=A-A(1-1/(1+kD^{s}))-A/(1-1/(1+kD^{s}))$
4	ADD4R	(9)	$SV=A-A(1-1/(1+kD^{sd}))-A/(1-1/(1+kD^{sp}))$
**Multiplicative**			
Amount & IV scaled			
3	MULTI3	(10)	$SV=A/({\left(1+kD\right)}^{s}*{\left(1+h\theta \right)}^{s})$
4	MULTI4	(11)	$SV=A/({\left(1+kD\right)}^{sd}*{\left(1+h\theta \right)}^{sp})$
Only IV scaled			
3	MULTI3R	(12)	$SV=A/((1+kD^{s})*(1+h\theta^{s}))$
4	MULTI4R	(13)	$SV=A/((1+kD^{sd})*(1+h\theta^{sp}))$

Several of these equations have been compared in previous research. Vanderveldt and colleagues [7] compared Eqs 5, 7, 10 and 11 in the domain of hypothetical gains and found that Eq 11 described the data best overall. However, adding additional free parameters typically leads to improved performance of a model. Thus, researchers often conduct statistical tests to determine whether the additional variance accounted for by adding free parameters is worth the reduced parsimony. Using incremental *F*-tests as the statistical criterion for model comparison, Vanderveldt et al. [7] continued to find that Eq 11 outperformed Eqs 5, 7 and 10 and, thus, the added variance accounted for with Eq 11 was worth the additional free parameters. But, researchers have not examined how well delayed lotteries are described by additive and multiplicative models where only the independent variables are exponentiated (Eqs 8, 9, 12, & 13). Past research with only delayed or only risky outcomes has been mixed as to whether the amount and independent variable scaled models describe behavior better than independent variable scaled models [22] or if independent variable scaled models outperform amount and independent variable scaled models [23].

The purpose of the two experiments reported in this paper was to determine how well subjective value with delayed lotteries are described by Eqs 4–13 using delay and probability ranges similar to previous research [7,21], and across the domains of gains and losses (Experiment 1). Second, we sought to determine how well subjective value with delayed lotteries is described by the same models but using a novel procedure that uses value equivalent delay and probability ranges (Experiment 2).

## Method

### Participants

Prior to the experiments, written informed consent was collected from every participant. For Experiment 1, 124 volunteers were recruited (98 female and 26 male; 27.12 ± 7.90 years old, mean age ± SD, ranging from 19 to 49 years of age) and randomly assigned to a gain or a loss condition (62 participants per condition). For Experiment 2, 132 volunteers were recruited (105 female and 27 male; 28.95 ± 8.60, mean age ± SD, ranging from 20 to 51 years of age) and randomly assigned to a gain or a loss condition (66 participants per condition). The participants in both experiments were students recruited from the university student pool and were awarded course credit for participation. All experimental procedures were approved by a local ethics committee of the first two authors (Faculty of Psychology, SWPS University of Social Sciences and Humanities).

### Procedure

The discounting task in Experiments 1 and 2 used an adaptive staircase choice algorithm [10,24,25]. Participants chose between two options: one option was an immediate and certain smaller amount (e.g., 100% probability of getting PLN 750 immediately); the second option was a delayed and probabilistic larger amount (e.g., 50% probability of getting PLN 1500 in 6 months). The position of each option was randomized to the left or right side of the computer screen.

In the gains conditions, if a participant chose the immediate reward, then the amount of the immediate reward decreased for the next choice. If the participant chose the larger, delayed and probabilistic option, then the amount of the immediate reward increased for the next choice. The size of the adjustment to the immediate option changed by an amount equal to *A/4n*, where *A* = the amount of the larger option and *n* = the *n*^th^ adjustment at the fixed delay-probability combination to the larger amount. For example, the first choice would ask the participant to choose between PLN 750 now and PLN 1500 at a given delay and probability of occurrence. The amount that the smaller option was adjusted after the first choice would be PLN 375 (*A/4n* = 1500/(4∙1) = 1500/4 = 375). Following the second choice, the smaller amount would adjust by PLN 187.5 (*A/4n* = 1500/(4∙2) = 187.5). This adjustment to the smaller amount continued for 6 total trials. In the loss conditions, the algorithm differed only in that if the immediate, certain, smaller amount was chosen, it would increase in the subsequent choice; and, if the delayed, probabilistic, larger amount was chosen, the smaller amount would decrease.

The adjusted amount of the smaller, immediate, certain option following the sixth trial was considered the indifference point for that specific delay and probability to the larger amount. In Experiment 1 a total of 25 indifference points were obtained for each participant from five delays (0, 1, 6, 24, and 60 months) crossed with five probabilities (1.00, 0.80, 0.40, 0.25, and 0.10). The larger amount was set to PLN 1500 (approximately 430 USD at the time of the study). Each participant completed a training session prior to the experimental procedures to familiarize them with the procedure. The training session presented choice options and adjusted the amount of the smaller option in an identical manner as the experimental procedure.

Experiment 2 included two stages: (1) delay and probability trade-off task (calibration), and the (2) multiple staircase procedure (similar to the procedure in Experiment 1). The trade-off task determined the probability equivalent to each delay. The calibration task was similar to the multiple-staircase procedure except we adjusted the probability of receiving a fixed payoff at a particular delay. Participants chose between two options. One option was PLN 1500 with an *x*% probability to be received. The second option was PLN 1500 to be received after a delay of *y*. In the first trial, *x* was set to 50% and then increased or decreased depending on the choice made by the participant. The probability adjusted by a similar multiple-staircase algorithm logic as Experiment 1 (i.e., 100%/4n; *p* adjusted by 25% after the 1^st^ choice, by 12.5% after the 2^nd^ choice, etc.). For participants in the gains group, choosing PLN 1500 with 50% probability led to a decrease in payoff probability; and, choosing the delayed option led to an increase in payoff probability. For participants in the loss group, choosing PLN 1500 with 50% probability led to an increase in payoff probability; and, choosing the delayed option led to a decrease in payoff probability. The procedure ended after the sixth choice for each delay with the final probability. The adjusted probability of the risky option following the sixth trial was considered the probability equivalent to that specific delay. This procedure was then repeated for each of 7 delays (0, 1, 6, 24, 60, 120, and 240 months).

Following the delay-probability trade-off task, participants in Experiment 2 completed a discounting task that was identical to Experiment 1 with one exception–the probabilities used for each participant were the probabilities identified through the delay-probability trade-off task. Participants made repeated choices between a smaller, immediate, and certain adjusting amount; and a larger, delayed, and fixed probabilistic amount. For example, if the delay-probability trade-off task found that a 78% probability of obtaining PLN 1500 was subjectively equal to receiving PLN 1500 in six months, then the discounting task would ask the participant to choose between a 100% probability of receiving PLN 750 now or a 78% probability of receiving PLN 1500 after 6 months. Similar to Experiment 1, participants completed a single training session for both the delay-probability trade-off task and the discounting task prior to the experimental tasks.

### Behavioral modeling

#### Model fitting

All procedures were performed in the R computational environment [26]. Models were fitted to the indifference points using nonlinear regression (i.e., a curve corresponding to each model was fitted to the observed indifference point values using maximum likelihood estimation).We implemented a genetic algorithm for parameter optimization using the R package *ga* [27,28]. This approach employs a stochastic optimization strategy inspired by principles of biological evolution and natural selection and is particularly efficient in avoiding local optima when applied to noisy environments and multiple-parameter models. Each model was fit to the indifference points obtained for each participant separately using maximum likelihood estimation to yield the best fitting parameters.

#### Model selection

For multi-model inference, we followed the guidelines by Wagenmakers & Farrel [29], with the exception that each model was fitted separately for each participant and that primary model comparisons were performed on the goodness-of-fit indices aggregated across participants. We used the Akaike Information Criterion with an additional term for bias correction (AICc [30,31]), and the Schwarz Bayesian Information Criterion (BIC [32,33]). Values of AICc or BIC obtained for each model were summed across participants to produce ∑AICc (or ∑BIC) and then transformed to ΔAICc (ΔBIC), which is the difference between ∑AICc (∑BIC) of given model and the minimum ∑AICc (∑_*i*_ BIC) observed in the candidate set [34,35].

In addition, we performed an overall comparison of all multiplicative models against the additive models. To that end, we computed normalized relative models likelihoods and then transformed the computed likelihoods to Akaike Weights (i.e., to probabilities that the model is the best in the candidate model set). Finally, we computed evidence ratios of the Akaike Weights corresponding to all multiplicative models against all additive models and *vice versa*. This procedure was performed for each participant separately and served to produce frequencies corresponding to the number of times the multiplicative models yielded greater evidence ratios than the additive models across participants. Equivalent procedures were performed based on the BIC metric by substituting BIC for AICc in corresponding computations.

#### Model validation

Following model selection, we validated the performance of the fitted models by inspecting their prediction errors. For each candidate model, in each participant, we derived the root mean square error (RMSE) from the difference between the observed and model-predicted indifference point values. We adopted this approach to assess model performance in absolute terms; that is, we assessed model performance by inspecting the error between the observed indifference point values and the values predicted by each candidate model for each indifference point for each participant. This was performed without correcting for model complexity (unlike model selection) to keep the following prediction error comparisons absolute. Pairwise comparisons of RMSE were then performed across all candidate models using Wilcoxon signed-rank test with a Bonferroni-Holm correction for multiple comparisons.

## Results

### Experiment 1: Arbitrary delay and probability values

In the domain of gains, ∑AICc or ∑BIC indicated that two models best described the obtained indifference points. The first best-performing model based on the lowest ∑AICc was a multiplicative four-parameter model where only the odds against and delay of the outcome were scaled by unique parameters (Eq 13; Table 2; ∑AICc = -2044.28, ΔAICc = 0). The best-performing model based on the lowest ∑BIC was a three-parameter model where delay and odds against were individually scaled using the same psychophysical scaling parameter (Eq 12; Table 2; ∑BIC = -1868.45, ΔBIC = 0). It should be noted that the three- and four-parameter multiplicative models were very close to each other for ∑AICc and ∑BIC for gains. In the domain of losses, the multiplicative three-parameter model where delay and odds against were individually scaled using the same psychophysical scaling parameter was again identified as the best description of subjective value using both goodness-of-fit indices (Eq 12; Table 2; ∑AICc = -1219.69, ΔAICc = 0 and ∑BIC = -1063.84, ΔBIC = 0).

**Table 2:** Results of Experiment 1. Model selection in the domain of gains and losses in Experiment 1. ADD indicates the basic structure of the equation is additive whereas MULTI indicates the basic structure of the equation is multiplicative. The number following ADD or MULTI indicates the number of free parameters in the model. Model names suffixed “R” indicates the scaling parameter is applied only to the delay or odds against variables rather than the entire denominator (see Table 1 for details).

Model	Equation	Number of parameters	Aggregated fit indices			
			Σ_*i*_ AIC_c_	Δ_*i*_ AIC_c_	Σ_*i*_ BIC	Δ_*i*_ BIC
Gains						
MULTI4R	13	4	-2044.28	0	-1866.00	2.46
MULTI3R	12	3	-2024.31	19.97	-1868.45	0
MULTI4	11	4	-1998.13	46.14	-1819.85	48.60
MULTI3	10	3	-1993.58	50.70	-1837.73	30.73
MULTI2	5	2	-1818.74	225.53	-1701.42	167.03
ADD3R	8	3	-830.25	1214.02	-674.40	1194.05
ADD3	6	3	-755.30	1288.98	-599.45	1269.00
ADD4R	9	4	-740.97	1303.31	-562.69	1305.76
ADD4	7	4	-732.20	1312.07	-553.92	1314.53
ADD2	4	2	-303.65	1740.63	-186.33	1682.13
Losses						
MULTI3R	12	3	-1219.69	0	-1063.84	0
MULTI3	10	3	-1201.46	18.23	-1045.61	18.23
MULTI4R	13	4	-1144.89	74.80	-966.61	97.23
MULTI4	11	4	-1093.73	125.97	-915.44	148.39
MULTI2	5	2	-930.76	288.94	-813.43	250.41
ADD3R	12	3	-775.90	443.79	-620.05	443.79
ADD3	6	3	-742.16	477.53	-586.31	477.53
ADD4R	9	4	-657.48	562.21	-479.20	584.64
ADD4	7	4	-636.86	582.83	-458.58	605.25
ADD2	4	2	-385.07	834.62	-267.75	796.09

We also compared the evidence ratios derived from AICc (ER_AICc_) and BIC (ER_BIC_) of all multiplicative model probabilities against the additive model probabilities in each participant separately. In gains, the evidence ratios favored the multiplicative models over the additive models in 95.16% and 96.77% of cases using ER_AICc_ and ER_BIC_, respectively. In losses, the evidence ratios favored the multiplicative models over the additive in 80.65% and 82.26% of cases using ER_AICc_ and ER_BIC_, respectively.

**Fig 1:**
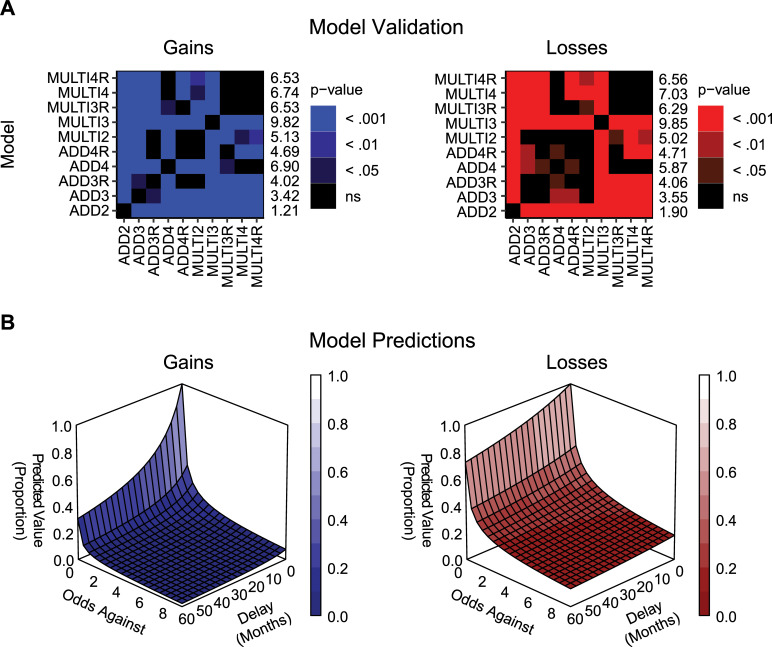
Results of Experiment 1. (**a**) Pairwise comparisons of model RMSE, derived from the difference between the observed and predicted indifference points values at each delay and odds against combination, in the domain of gains or losses for each model and participant. Numbers on the right of the panels represent mean ranks of RMSE. The higher the rank, the lesser was the spread of prediction errors in fits of given model (lower RMSE). The numbers to the right of each plot correspond to mean rank across participants for that model. (**b**) Values predicted by the three-parameter multiplicative model with the amount, the odds against and delay of the outcome scaled (MULTI3, Eq 10) as a function of outcome delay and odds against in gains or losses. Median parameter estimates for the MULTI3 model were used to produce each plot. Median values were: *k* = 0.10, *h* = 7.28, *s* = 0.61 in the domain of gains and *k* = 0.02, *h* = 6.99, *s* = 0.41.

Thirdly, we validated the performance of each model. Pairwise comparisons of the RMSE values obtained using each model (Fig 1A) for each participant showed that the three-parameter multiplicative model with the amount as well as the odds against and delay of the outcome scaled (Eq 10), produced lower RMSE than other candidate models in the domain of gains (mean rank = 9.82) and in losses (mean rank = 9.85). The comparisons against all other candidate models were highly significant (*p* <. 001 in all cases). The two-parameter additive model produced the highest RMSE in gains (mean rank = 1.21) and in losses (mean rank = 1.90), which differed significantly against that produced by any other candidate model (*p* < .001).

Finally we investigated an interaction between delay and probability. Visual analysis of plots presented in Fig 1B supports an interaction between delay and probability in discounting of delayed lotteries. That is, the shape of the delay discounting curve changed depending on the odds against receiving the outcome; and the shape of the probability discounting curve changed depending on the delay to receiving the outcome. Similar to previous research [7,21], delay had a larger impact on the subjective value of the reward at lower odds against compared to higher odds against. Odds against had a slightly larger impact on the subjective value of the reward at shorter delays compared to longer delays. An interaction between probability and delay was also supported statistically through an ANOVA performed on individual indifference points in the domain of gains (*F*(16, 46) = 10.49; *p* < .001; η_p_^2^ = .78) and losses (*F*(16, 46) = 3.12; *p* = .001; η_p_^2^ = .52).

### Experiment 2: Individual delay-probability tradeoffs

**Fig 2:**
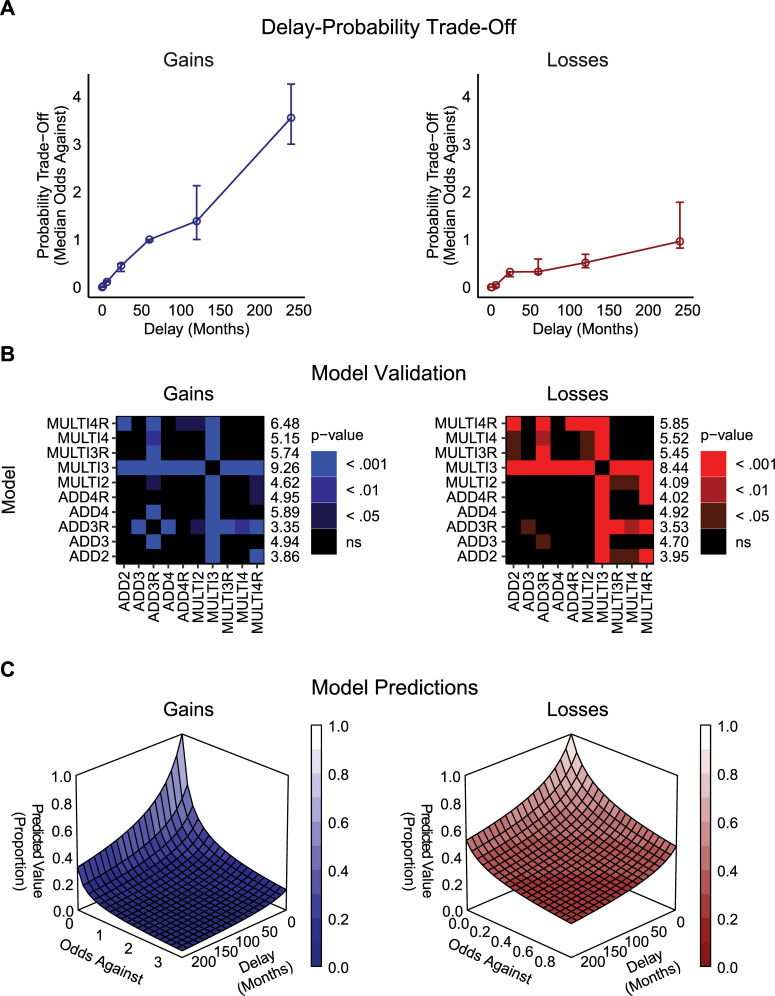
Results of Experiment 2. (**a**) Median delay and probability trade-off values obtained in the calibration task in the domain of gains or losses; error bars represent bootstrapped 95% confidence intervals (CI) of the median. (**b**) Pairwise comparisons of model RMSE, derived from the difference between the observed and predicted indifference points values at each delay and odds against combination, in the domain of gains or losses for each model and participant. Numbers on the right of the panels represent mean ranks of RMSE. The higher the rank, the lesser was the spread of prediction errors in fits of given model (lower RMSE). (**c**) Values predicted by the three-parameter multiplicative model with the amount as well as the odds against and delay of the outcome scaled (MULTI3, Eq 10) as a function of outcome delay and odds against in gains or losses. Used were median parameter estimates for the MULTI3 model, which were: *k* = 0.02, *h* = 5.93, *s* = 0.61 in the domain of gains and *k* = 0.03, *h* = 10.10, *s* = 0.31.

Fig 2A illustrates the results of the delay-probability trade-off task in Experiment 2. Visually, the odds-against equivalent increased (probability equivalent decreased) as the delay of the outcome increased, with the decrease greater in the domain of gains compared to losses. For the gains and losses groups, the probability equivalent of a delayed outcome differed significantly depending on the length of the delay (Friedman test, for gains: *Q* = 322.87; *p* < .001; and losses: *Q* = 188.48; *p* < .001). Specifically, the probability equivalents decreased as the delay increased for gains (Fig 2A, left panel), and for losses (Fig 2A, right panel). Pairwise comparisons of gains and losses at each delay using Mann-Whitney U tests indicated that the probability equivalents decreased significantly more with increased delay to gains compared to increased delay to losses at 60, 120, and 240 months (60 months, *U* = 1747.50, *p* = 0.049; 120 months, *U* = 1720.00, *p* = 0.037; 240 months, *U* = 1599.50, *p* = 0.008).

We also compared how well the models described indifference points involving delayed lotteries when using the delay-probability equivalents identified in the delay-probability trade-off task. In the domain of gains a multiplicative three-parameter model (odds against and delay of the outcome scaled; Eq 12 was uniformly indicated as the best description of the obtained indifference points using ∑AICc and ∑BIC indices (Table 3; ∑AICc = -1089.45, ΔAICc = 0; ∑BIC = -1628.16, ΔBIC = 0). The same model (Eq 12) was also indicated as the best description of indifference points in the domain of losses using ∑AICc and ∑BIC indices (Table 3; ∑AICc = -1474.18, ΔAICc = 0; ∑BIC = -2012.89, ΔBIC = 0).

**Table 3:** Results of Experiment 2. Model selection in the domain of gains and losses in Experiment 2. ADD indicates the basic structure of the equation is additive whereas MULTI indicates the basic structure of the equation is multiplicative. The number following ADD or MULTI indicates the number of free parameters in the model. Model names suffixed “R” indicates the scaling parameter is applied only to the delay or odds against variables rather than the entire denominator (see Table 1 for details).

Model	Equation	Number of parameters	Aggregated fit indices			
			Σ_*i*_ AIC_c_	Δ_*i*_ AIC_c_	Σ_*i*_ BIC	Δ_*i*_ BIC
Gains						
MULTI3R	12	3	-1089.45	0	-1628.16	0
MULTI2	5	2	-1063.74	25.70	-1268.88	359.27
MULTI3	10	3	-882.38	207.07	-1421.09	207.07
ADD2	4	2	-761.42	328.02	-966.56	661.59
ADD3R	8	3	-718.18	371.26	-1256.89	371.26
ADD3	6	3	-261.79	827.65	-800.50	827.65
MULTI4R	13	4	-251.38	838.07	-1585.66	42.50
MULTI4	11	4	-33.78	1055.67	-1368.06	260.10
ADD4R	13	4	-19.34	1070.10	-1353.62	274.53
ADD4	7	4	112.52	1201.97	-1221.76	406.40
Losses						
MULTI3R	12	3	-1474.18	0	-2012.89	0
MULTI2	5	2	-1259.47	214.71	-1464.61	548.28
MULTI3	10	3	-1191.00	283.18	-1729.71	283.18
ADD3R		3	-1127.34	346.84	-1666.05	346.84
ADD2	4	2	-1006.50	467.68	-1211.64	801.25
ADD3	6	3	-892.04	582.14	-1430.75	582.14
MULTI4R	9	4	-575.42	898.77	-1909.70	103.20
MULTI4	11	4	-556.76	917.42	-1891.04	121.85
ADD4R	9	4	-424.91	1049.27	-1759.19	253.70
ADD4	7	4	-366.02	1108.17	-1700.30	312.60

As in Experiment 1, we compared the evidence ratios derived from AICc (ER_AICc_) and BIC (ER_BIC_) of all multiplicative model probabilities against all additive model probabilities. For gains, evidence ratios favored the multiplicative models in 81.82% and 83.33% of all cases using ER_AICc_ and ER_BIC_, respectively. In losses, the evidence ratios favored the multiplicative models in 72.73% and 65.15% of all cases using ER_AICc_ and ER_BIC_, respectively.

Similar to Experiment 1, we also validated the performance of each model through pairwise comparison of the RMSE values obtained for each participant using each model. A three-parameter multiplicative model (amount, odds against, and delay scaled; Eq 10) produced the lowest RMSE values across participants in the domain of gains (mean rank = 9.26; Fig 2B, left panel) and losses (mean rank = 8.44; Fig 2B, right panel), which significantly differed against all other models (*p* < .001). No other model achieved significant differences in all model pairings. The overall shape of the discounting curves predicted by this model is shown in Fig 2C.

## Discussion

Human choice often involves delayed outcomes that are also uncertain to occur (i.e., delayed lotteries). We conducted two experiments with participants randomly assigned to a gain or a loss group. The experimental procedures in both studies asked the participants to make a series of choices between a smaller-sooner-certain option and a larger-later-uncertain option. In Experiment 1, the delay and probability associated with the larger outcome were systematically varied to obtain 25 indifference points spanning delays of immediate to 5 years and spanning probabilities of 1.00 to 0.10. In Experiment 2, participants first completed a calibration task to determine the probability of receiving PLN 1500 that was equivalent to receiving PLN 1500 at each of 5 delays spanning immediate to 20 years. Participants then completed a second task where they chose between a smaller-sooner-certain option and a larger-later-uncertain option where the delay and risk associated with the larger option were the probability-delay equivalents determined in the first task. We used nonlinear regression to fit 10 different candidate discounting models that describe choice with delayed lotteries. Each model differed in the number of free parameters; assumptions about psychophysical scaling processes of amount, delay, and risk; and assumptions about whether delay and probability interact to influence outcome value.

In both experiments, AICc and BIC indicate the multiplicative models described data better than the additive models with the strongest support for Eq 12. The structure of Eq 12 suggests that delay and probability interact to influence the final subjective value of delayed lotteries for gains and losses, and a single psychophysical scaling parameter can be used for both delay and probability. However, to evaluate model performance in more practical terms, we also compared their prediction errors across participants (i.e., the errors in predicting the actual indifference points by each model). These comparisons uniformly indicated the three-parameter multiplicative solution in which only the discounting factors are scaled by a single parameter (Eq 10). This approach allowed us to select the model in the candidate set that best predicted individual subjective value. Such approach in model validation seems useful not only in selecting the most practical model, but also as an alternative approach to the assumption-heavy nature of the AICc and BIC model selection criteria [36].

A seemingly straightforward quantitative explanation exists as to why the information theoretic approaches and the absolute prediction error approaches suggested different best models. The information-theoretic approaches account for model complexity while measuring the goodness of fit. The absolute measure relies solely on the goodness of fit. Thus, it makes sense that including an extra free-parameter results in better fits overall leading to its selection in absolute terms, while penalizing for the added complexity reduces its ranking below other, less complex models. The difference in model ranking outcomes that result from using the different approaches suggests how closely the different models were in their overall ability to describe the observed data. At present, this might suggest that the function of using the quantitative models would determine which of these two models a researcher chooses [37].

Because the multiplicative nature of delay and probability in influencing choice had been observed in the domain of gains in three prior experiments [7,21], and one in the domain of losses [21]. Our results further supports that delay and probability interact to influence choice in delayed lotteries. This suggests that the impact of risk on subjective value will diminish as delay increases. Similarly, the impact of delay on subjective value will diminish as risk increases.

Our results also add to a growing literature that suggest a single quantitative model can describe behavior with delayed outcomes, risky outcomes, and risky-delayed outcomes (i.e., delayed lotteries). But, this does not mean that delay and probability are reducible to one another. Previous research sought to determine if risk was more fundamental (i.e., that delay could be reduced to an risk equivalent [38–40]), or if delay was more fundamental (i.e., that probability could be reduced to a delay equivalent [1,8,41]). But, several differences between choice in risky and intertemporal domains have been observed that are incompatible with a reducibility hypothesis. For example, the magnitude effect refers to a common observation that large delayed gains are discounted less steeply than small delayed gains [42–45]. However, this effect reverses for probabilistic outcomes as large risky gains are discounted more than small risky gains [43,44]. Other observations that contradict a reducibility hypothesis are a lack of correlation between delay and probability discounting [46–48] and a different impact of gains and losses on discounting delayed outcomes compared to risky outcomes [49]. Thus, delay and probability seem to be unique outcome characteristics that interact to influence choice.

Psychophysical scaling parameters are often added to discounting data from human participants [15–17,22]. However, an open question has been whether the psychophysical scaling parameter should exponentiate the entire denominator in discounting equations (which scales amount and the independent variable simultaneously) or if the scaling parameter should exponentiate only the independent variable (delay or probability). Some research has observed that scaling the entire denominator describes choice better [22] whereas other research has observed that scaling only the independent variable describes choice better [23]. However, these comparisons were only used with delayed or risky outcomes–not with delayed lotteries. The results from our model comparisons with delayed lotteries indicated that scaling is important. The open question is whether only the independent variables should be scaled or scaling should be applied to the entire denominator.

Including psychophysical scaling parameters in descriptions of delayed lotteries raises another question. Should researchers use a single parameter to scale both delay and odds against? Or, should delay and odds against be scaled independently from the other? Stated differently, is the descriptive power gained from having two scaling parameters worth the reduced parsimony that accompanies an added free parameter. The results of our model comparisons indicated that the descriptive power gained from using two, separate psychophysical scaling parameters (i.e., one for delay and one for probability) was not worth the increased model complexity. These results contrast previous research comparing 3 and 4 parameter multiplicative models [7]. Although Vanderveldt and colleagues tested only models in which the entire denominator was scaled, they found that having a separate scaling parameter for delay and for probability was worth the added complexity.

It is worth acknowledging some differences between the analytical approach used in this study, and previous reports. Vanderveldt and colleagues [7] used an extra-sum-of-squares *F*-test to compare models that were separately fit to group and median indifference points. We used a corrected Akaike’s Information Criterion (AIC_c_), BIC, and RMSE to compare models fit using maximum likelihood estimation, with the models fitted separately for each participant and goodness-of-fit metrics aggregated across participants. Future research could determine the contexts in which each approach is most appropriate and whether each approach leads to a different understanding of preferences with delayed lotteries.

As noted by several authors [7,21], one reason probability may have been found to be more influential in past research are the ranges of delays and probabilities chosen for the experiment. Specifically, the probabilities assessed in past research have remained relatively constant and have encompassed the full range of a bounded scale (0 < *p* < 1). In contrast, the delays assessed in past research have varied in terms of the longest delay used and therefore past research has used different segments of an unbounded scale (0 < *d* < ∞). Furthermore, a robust finding in discounting research is that different people show different rates of discounting [50]. Thus, it seems reasonable that delay and probability may influence outcome values differently for different people. One way to examine whether delay or probability influences outcome value more would be to determine the delays and probabilities wherein outcome value is equivalent for each person. These delay and probability ranges could then be used in discounting tasks where outcomes are both delayed and probabilistic to allow for comparison of value equivalent ranges of delay and probability.

The use of delay and probability ranges of potentially unequivalent influence raises a potential confound in previous research on delayed lotteries. We sought to control for that confound in Experiment 2 by first determining the probability equivalents of various delays for each participant using a calibration task. The results from the calibration suggested that the longest delay used in previous research of 60 months (5 years) [7,21] corresponded to approximately a 50% probability of a gain, or a 76% of incurring a loss. Even the maximum delay of 20 years that was used in Experiment 2 led to corresponding probabilities of 22% for gains and 51% for losses. The data from Experiment 2 suggest that the probabilities used in previous research covered a substantially greater range of influence on outcome value compared to the delays used and may explain why previous researchers observed that probability influenced outcomes more than delay [7,21,51]. Nevertheless, the best fitted models were similar across Experiments 1 and 2 despite the differences in subjective influence inhereit to the different ranges of delay and probability that were used.

A potential limitation to this research is that we used a select set of models from the discounting research paradigm based on behavior analytic theory. The purpose of this experiment was to compare descriptions of choice at both the individual and group level where a set of indifference points were obtained for each participant across a parametric range of delays and probabilities. The models tested are well-established descriptions of human and nonhuman discounting at the individual level. However, other approaches have been used to describe choice between two delayed and risky outcomes. For example, some researchers have used models derived from prospect theory that describe the proportion of participants in a group that choose one outcome over another [52,53]. Other researchers have used heuristic, attribute-based models that rely on different theoretical assumptions for decision-making and focus on describing out-of-sample choice [54,55]. These alternative approaches and models for describing delayed lotteries were not tested in this experiment. Future research aimed primarily at describing choice at the group level could consider these models in addition to those tested in the current experiment.

Another limitation is that we used hypothetical consequences. There is general consensus that procedures using hypothetical and real outcomes provide comparable results in discounting research [13,56,57]. However, this has not been tested for choice with delayed lotteries. Future research could examine real and hypothetical delayed lotteries to determine the extent that choice is comparable. Such research may allow researchers to better understand the impact of delayed lotteries on everyday decisions and the range of delays and probabilities relevant for researchers to examine in non-laboratory settings.

Understanding daily choices that carry social importance may also benefit from future research that examines different commodities. To date, research on delayed lotteries has used monetary outcomes. This research may be helpful with financial decision-making where risk and delay have been incorporated in sophisticated ways [58,59]. But, past research also suggests that discounting changes depending on the commodity under consideration [60,61], and that discounting with one commodity may not be related to discounting of different commodities [62–66] c.f. [25,60,67,68]. It is relatively unknown how risk and delay are incorporated in decisions where choice involves delayed lotteries of non-monetary commodities (e.g., addiction, health behavior, medication adherence). Parametric analyses of delayed lotteries across varied commodities may improve description and prediction of behavior in more complex, everyday situations.

## Conclusions

We compared 10 quantitative discounting models that described subjective value with outcomes that were delayed and risky (i.e., delayed lotteries). The results suggest subjective value was described best by a three-parameter multiplicative discounting model. Information criteria indicated delay and odds against should each be raised to the same psychophysical scaling parameter (*s*) and absolute measures of fit indicate the entire delay and probability denominators in hyperboloid discounting equations should be raised to the same psychophysical scaling parameters. The results of model comparisons for describing subjective value with delayed lotteries were similar for gains and losses. Finally, the results of model comparisons were similar when using delay and probability ranges of equivalent influence.
